# Burden of virologic failure, and its determinants among HIV positive children in East Africa: a systematic review and meta-analysis

**DOI:** 10.4314/ahs.v24i3.2

**Published:** 2024-09

**Authors:** Bogale Chekole Temere, Agerie Aynalem Mewahegn, Bitew Tefera Zewudie, Fisha Alebel GebreEyesus, Haymanot Abebe, Ahmed Nuru, Tamene Fetene, Shegaw Tesfa, Tadesse Tsehay Tarekegn, Amare Kassaw, Wubet Alebachew, Belete Gelaw Walle, Shegaw Geze Tenaw, Yibeltal Mesfin, Muche Argaw, Daniel Tsega

**Affiliations:** 1 Department of Nursing, College of Medicine and Health Science, Wolkite University Southwest Ethiopia; 2 Department of Pediatric Nursing, College of Medicine and Health Science, Debre Tabor University, Northwest Ethiopia; 3 Department of Maternal & neonatal nursing, College of Medicine and Health Science, Debre Tabor University, NorthWest Ethiopia; 4 Department of Pediatric Nursing, College of Medicine and Health Science, Wolaita Sodo University, SouthWest Ethiopia; 5 Department of Midwifery, College of Medicine and Health Science, Wolkite University Southwest Ethiopia

**Keywords:** HIV, virologic failure, adherence, viral load suppression

## Abstract

**Background:**

There are many studies regarding the prevalence of Virologic failure and its associated factors in East Africa, but they are inconclusive. In this study, the prevalence of Virologic failure and its associated factors in east Africa were determined.

**Methods:**

Published articles that were conducted in English language were prepared based on preferred reporting items for systematic reviews and meta-analysis (PRISMA) statement. Web of Science, PubMed, and Google Scholar were explored to find out the articles. STATA Version 14 software was used for computing the pooled estimates of both the prevalence of virologic failure and associated factors. The pooled estimates were computed using both random and fixed effect models. Results were presented by using the randomand fixed effect models. . The pooled estimates were presented with 95% CI. Publication bias was assessed by looking the symmetry in the funnel plot.

**Result:**

The pooled prevalence of virologic failure was, 25.90 (19.49, 32.32, p ≤ 0:001) in the random effect model. Four factors, poor adherence (2.91 (Pooled odds ratio(POR): 2.91, 95%CI: 1.93, 4.40), being male (1.17 (POR: 1.17, 95%CI: 1.08, 1.27)), wasting as an under nutrition (3.1 (POR:3.1, 95%CI: 1.63, 5.95), and Nevirapine(NVP) based regimen (2.76 (POR: 2.76 (1.65,95%CI: 4.62)) were found to be independent predictors of virologic failure.

**Conclusion:**

The prevalence of virologic failure in East Africa is remarkably high. Four factors were found to be the determining factors Different strategies must be designed to address (poor adherence, being male, wasting as an under nutrition, and NVP based regimen).

## Background

Virologic failure is considered when the viral load above or equal to 1000 copies/ml under ART based on two consecutive VL results 3 months apart, with adherence support following the first viral load test after at least 6 months of ART^1^.

Globally, a total of 1.8 million children(up to age 18 years) living with HIV and an estimated 110,000 AIDS-related deaths were reported^2^.

Sub-Saharan Africa and its sub region shares the higher global burden of HIV- infection^3^. There were evidences of Virologic failure from different countries across the globe. There were persistent disparities in the level of Virologic suppression, across countries' in Europe^4^. The prevalence of Virologic failure in Asian HIV positive children was17.9%^5^.

Clinical, socioeconomic and factors that may be associated with Virologic failure vary between and within racial and ethnic groups^6^.

It is known that the goal of antiretroviral therapy is to suppress viral replication and reduce HIV related mortality among positive children. The World Health Organization (WHO) recommends routine viral load monitoring for all patients on ART. Since, Virologic failure believed to be early and accurate indicator for treatment failure during monitoring people on ART^7^.

However, there are studies conducted in Ethiopia and other East African countries, the findings were inconsistent and non-conclusive. So, the aims of this review were to estimate the pooled prevalence of Virologic failure and associated factors among HIV positive children in East Africa.

## Methods

### Eligibility Criteria and Information Sources

In this systematic review and meta-analysis, studies conducted in East Africa with an objective of assessing the prevalence of Virologic failure among HIV positive children were included. The studies were assessed using study area, title, abstract, and full texts prior to inclusion in this study. This study is prepared based on preferred reporting items for systematic reviews and meta-analysis (PRISMA) statement^8^. In the current study, published articles that were conducted in English language were explored and included. The EndNote X5 reference manager was used to manage retrieved articles.

### Search Strategy and Selection of Studies

Electronic databases and reference lists of articles were explored by two investigators. Web of Science, PubMed, and Google Scholar were explored to find out the articles. Searching was conducted using key terms: children and adolescents as a population, factors, risk factors, and determinants as an exposure, prevalence, virologic failure, and treatment failure as an outcome, cohort studies, cross-sectional, and observational surveys as a study design, and East Africa, and East African countries as a study location. The Boolean search operators such as “OR” and “AND” were used during the searching process.

### Data Extraction Process

The structured data extraction checklist was used to collect information by two authors. Name of the author(s), publication year, and region as a study country, sample sizes, and the prevalence of virologic failure in proportion were extracted for this study.

### Quality Assessment of Studies

The two writers independently evaluated the included studies' merits using the National Institutes of Health (NIH) quality Assessment method to ascertain the research' caliber. As a result, each question received a “yes,” “no,” “cannot determine,” “not applicable,” or “not reported” response^9^.

### Statistical Methods and Analysis

In the present meta-analysis, STATA Version 14 software was used for computing the pooled 7 estimates of both the prevalence of virologic failure and its associated factors. The pooled estimates were computed using both random and fixed effect models. Results were presented by using the random effect models. Subgroup analyses were performed using the country as a region. The pooled estimates of factors were also presented separately. The pooled estimates were presented with 95CI. Meta-analyses were presented using forest plot, summary tables, and texts.

### Publication Bias and Heterogeneity

Publication bias was assessed by looking at the asymmetry of the funnel plot. Heterogeneities among studies were explored using forest plots and I2 test. The sources of possible significant heterogeneities were explored through subgroup analyses and sensitivity analysis.

## Results

### Selection of Eligible Studies

**Figure 1:**
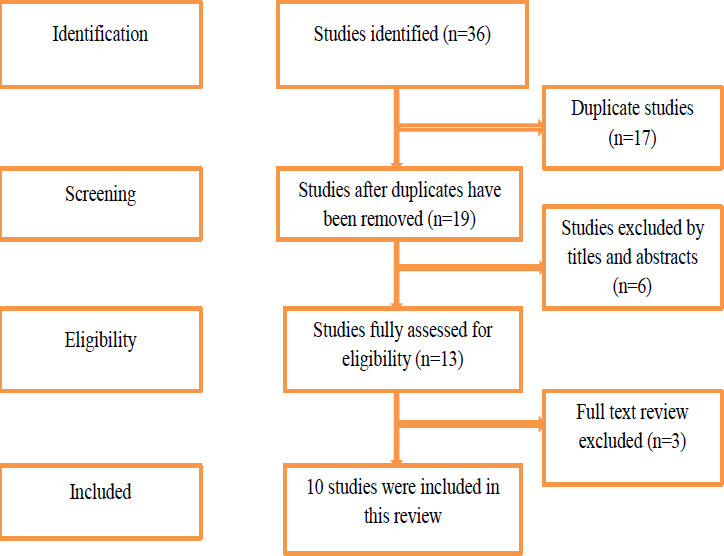
PRISMA flow chart showing the literature search processes for systematic and meta-analysis of virologic failure among children in East Africa(20)

As an initial search, we found 36 studies, retrieved from electronic databases (PUBMED, HINARI & others). From these studies 17 were duplicated files and 6 studies were removed after screening their titles and abstracts. The full texts of 13 studies were reviewed. Finally, 10 studies (10-19) were included in the final analysis of this systematic review and meta-analysis (Fig 1).

### Characteristics of the Studies

From the studies included in this study 7 of them were cross-sectional and 3 of them were cohort studies. Regarding the study region, three studies from Ethiopia(11, 13), three studies from Tanzania(12, 17, 19), two studies from Uganda(15, 18) and two studies from Kenya(10, 16) were included (Table 1).

**Table 1:** Descriptive summary of seven studies in systemic and meta-analysis of virologic failure among children in East Africa

Authors	Year	Region	Sample size	Cases	Study design	
Gelaw et al	2020	Ethiopia	424	59	cross-sectional	94.10%
Bayleyegn B et al	2021	Ethiopia	253	49	cross-sectional	100.00%
Tadesse BT et al	2020	Ethiopia	484	72	cross-sectional	100.00%
Kabogo JM et al	2013	Kenya	146	35	cohort	100%
Emmett SD et al	2010	Tanzania	206	65	cross-sectional	100.00%
Martelli G et al	2019	Tanzania	300	102	cross-sectional	100.00%
Tabb Zj et al	2015	Tanzania	280	91	cross-sectional	81%
Natukunda et al	2015	Uganda	238	69	cross-sectional	84.00%
Kamya MR et al	2007	Uganda	250	58	cohort	89%
Kadima J et al	2018	Kenya	1190	444	cohort	100.00%

### The Pooled prevalence of virologic failure

**Figure 2:**
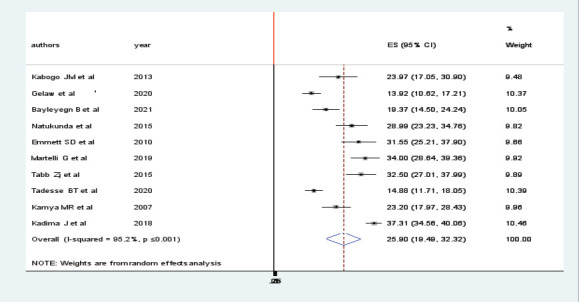
Forest plot in the pooled prevalence of virologic failure among HIV positive children in East Africa

The prevalence of viral failure was lowest (13.92%) and highest (37.31%) in studies done in Ethiopia and Kenya, respectively. In the random effect model, the pooled prevalence of Virologic failure was 25.90 (19.49, 32.32, p 0:001) (Fig 2).

### Subgroup analysis of prevalence of virologic failure

**Figure 3:**
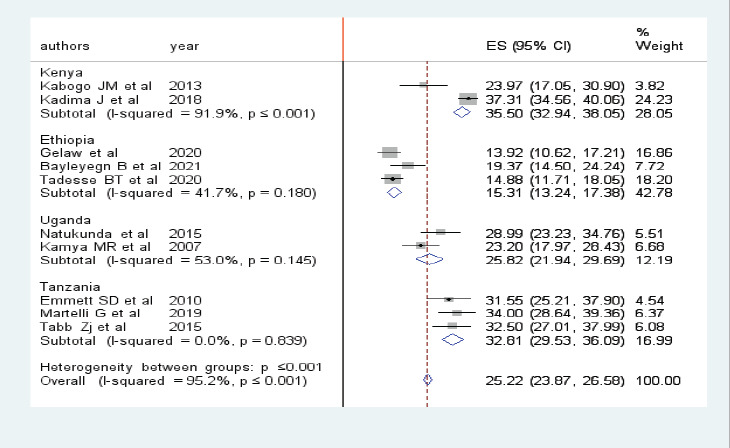
Forest plot showing the subgroup analysis of the pooled prevalence of virologic failure among HIV positive children in East Africa

Subgroup analysis by region was performed to compare the prevalence of virologic failure among children across countries in East Africa. Accordingly, the highest prevalence was observed in Kenya 35.50 (32.94, 38.05) followed by Tanzania 32.81 (29.53, 36.09) and the lowest prevalence 15.31 (13.24, 17.38) was observed in Ethiopia (fig 3).

### Heterogeneity and publication bias

**Figure 4:**
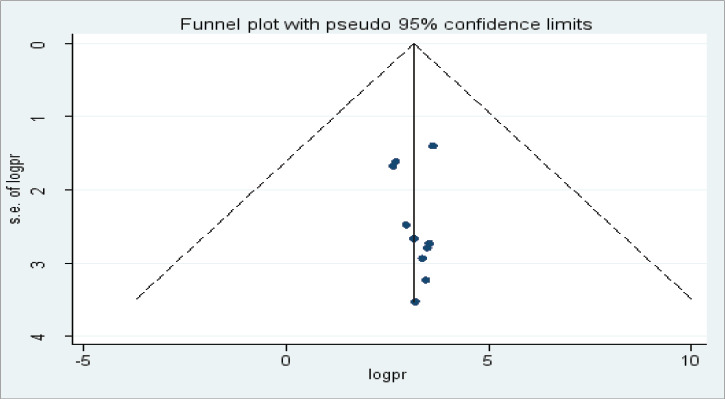
Funnel plot showing publication bias among studies used to compute the prevalence of virologic failure among HIV positive children in East Africa. Sensitivity analysis was also done and it was found that no single study affected the pooled prevalence (Fig 5)

We assessed for the presence of publication bias among the studies included in the review by using a funnel plot. There was a symmetrical distribution of the included studies through visual inspection, which indicates there was no potential publication bias (Fig 4).

**Figure 5:**
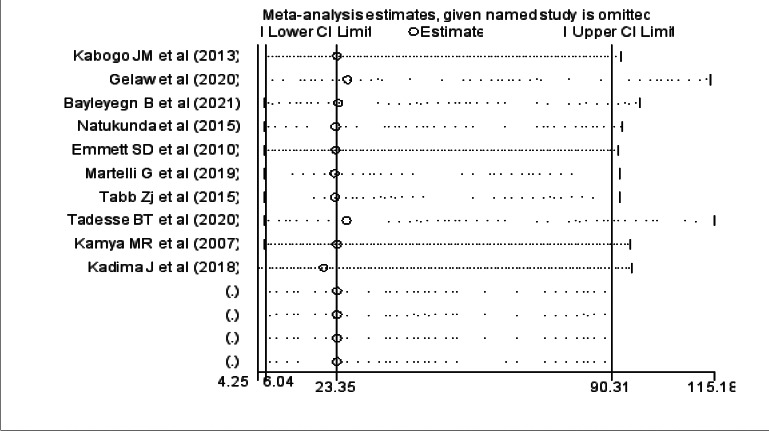
Sensitivity analysis of studies used to compute the prevalence of virologic failure among HIV positive children in East Africa

### Factors associated with Virologic failure among children in East Africa

Seven studies^11^-^14^, ^16^, ^18^, ^19^ were included in the analysis of the factors of virologic failure among children in East Africa. Four factors were included in this meta-analysis: poor adherence, being male, wasting as an under nutrition, and NVP based regimen. All of these factors were independent predictors of virologic failure. Male children were 1.17 (POR: 1.17, 95%CI: 1.08, 1.27) times more likely to develop virologic failure as compared to females. The odds of developing virologic failure among children with poor ART adherence were 2.91 (POR: 2.91,95%CI: 1.93, 4.40) times higher as compared to those with good adherence. The odds of developing virologic failure among children who were on NVP based regimen 2.76 (POR: 2.76 (1.65,95%CI: 4.62) times higher. Severely wasted children were 3.1 (POR: 3.1, 95%CI: 1.63, 5.95) times more likely to develop virologic failure.

## Discussion

The pooled prevalence of virologic failure was computed using both fixed and random effect models. But, the final estimates were reported using the random effect model, since there was remarkable heterogeneity among studies (I2, 95.2%, p ≤ 0:001). The pooled prevalence of virologic failure in East Africa was 25.9%. Due to high heterogeneity among the included studies, these pooled estimates were presented with a random effect model. In this study, sub- group analyses were performed to estimate the pooled prevalence of virologic failure for countries in East Africa. The pooled prevalence of virologic failure in Ethiopia, Tanzania, Uganda, and Kenya were 15.31%, 32.81%, 25.82%, & 35.5% respectively. Finally, sensitivity analysis was performed, there was observed heterogeneities between the included studies. The possible source of heterogeneity might be the presence of variation in the prevalence of virologic failue in East African countries. From this finding, the pooled prevalence of virologic failure is remarkably higher in Kenya. This could be due to the high burden of poor ART adherence in the country. Regarding the factors, Virologic failure in male children was significantly higher as compared to females. This finding is supported by previous studies^21^, ^22^. This might be due to physiological differences. Similarly, the odds of virologic failure in children with poor ART adherence are considerably higher than those with good adherence. this is supported by previous studies^23^. The possible explanation for this is due to poor adherence patients will develop drug resistance, Which will in turn cause the development of virologic failure^24^. Severe wasting was also an independent factor of virologic failure. The possible rationale might be the effect of malnutrition on the bio availability of ART drugs^25^. Besides this, NVP based regimen was found to have a significant association with virologic failure and it is supported by a previous studies^26^-^28^. The other possible rationales for those identified factors need to be explored by future researches.

However, this study has strength in finding all possible articles; the presence of scant studies in the two countries could mask some other factors of virologic failure in East Africa. It had a risk of bias, incomplete retrieval of identifying research and reporting bias. Since most of the studies included in this review were cross-sectional, it is impossible to establish cause and effect relationship. The other limitation in this systematic review was all countries of East Africa did not include it, so it might lack representativeness of the eastern African countries. Since the number of studies included in our sub-group analysis was small, the precision of the estimate might be reduced.

## Conclusion and recommendations

The prevalence of Virologic failure in East Africa is remarkably high. Four factors (poor adherence, being male, wasting as an under nutrition, and NVP based regimen) were found to be the determining factors. Different strategies must be designed by the nations of east Africa to address these factors to decrease the burden of Virologic failure in East Africa. We recommend prospective longitudinal researches to be done in the East African countries to identify all possible factors.

## Figures and Tables

**Table 2:** Factors associated with virologic failure among HIV positive children in East Africa

Factors	Included studies	OR (95%CI)	POR (95%CI)	Heterogeneity
**Poor adherence**	Tadesse BT et al	3.9(1.1,13.4)		
	Martelli G et al	3.22(1.87,5.56)		
	Gelaw et al	2.19(1.05,4.57)	2.91(1.93, 4.40)*	I^2^=0.92, p = 0.632
**NVP based regimen**	Kamya MR et al	2.46(1.23, 4.9)		
	Martelli G et al	3.19(1.47,6.89)	2.76(1.65, 4.62)*	I^2^=0.24, p = 0.625
**Sex (Male)**	Kadima J et al	2.2(1.3,3.8)		
	Jorojick et al	1.14(1.04,1.23)		
	Kamya MR et al	2.44(1.2,4.93)	1.17(1.08, 1.27)*	I^2^=9.85, p = 0.007
**Severe wasting**	Bayleyegn B et al	3.02(1.19,7.67)		
	Kadima J et al	3.2(1.3,7.9)	3.1(1.63, 5.95)*	I^2^=001, p = 0.930

**CI: confidence interval, OR: odds ratio, NVP: Nevirapine, POR: pooled odds ratio**

## Data Availability

The datasets analyzed during the current study are available from the corresponding author on reasonable request and will be attached to the editorial office when requested at any time.

## Abbreviations

CI: Confidence interval
HIV: Human immunodeficiency virus
OR: Odds ratio
POR: Pooled odds ratio
UNAIDS: United Nations Programme on HIV/AIDS
